# The discovery of a data-driven causal diagram of sport participation in children and adolescents with heart disease: a pilot study

**DOI:** 10.3389/fcvm.2023.1247122

**Published:** 2023-11-21

**Authors:** Jakub S. Gąsior, Marcel Młyńczak, Craig A. Williams, Aleksandra Popłonyk, Daria Kowalska, Paulina Giezek, Bożena Werner

**Affiliations:** ^1^ Department of Pediatric Cardiology and General Pediatrics, Medical University of Warsaw, Warsaw, Poland; ^2^ Faculty of Mechatronics, Institute of Metrology and Biomedical Engineering, Warsaw University of Technology, Warsaw, Poland; ^3^ Public Health and Sports Sciences Department, Children’s Health and Exercise Research Centre, Faculty of Health and Life Sciences, University of Exeter Medical School, University of Exeter, Exeter, United Kingdom; ^4^ Department of Physiotherapy, Medical University of Warsaw, Warsaw, Poland

**Keywords:** causal diagram, sport, children, adolescents, heart disease, heart defects, pediatric patients

## Abstract

The interventions aimed at improving the levels of physical activity (PA) in children and adolescents diagnosed with heart disease did not produce the expected outcomes. Safe participation in sport activities proposed based on actual recommendations could be a solution to promote PA in this population. The aims of this study were to discover a causal diagram of sport participation in children and youth with heart disease and establish the factors that affect and are affected thereof through the use of questionnaires. Furthermore, the study aims to qualitatively assess the reliability of the constructed diagram in comparison with existing medical knowledge. The Greedy Fast Causal Inference method was employed to conduct a data-driven search of the directed acyclic graph that represents the causal relationships within the provided observational data. This causal discovery was performed using the Tetrad software. The analysis involved a cohort of 121 Caucasian patients (50 females) diagnosed with heart disease. The age range of the patients included in the study was 8–17 years. The study findings indicate that the participants engaged in sports presented significantly higher values of health-related quality of life (QoL) and motives for participating in physical and leisure activities. Age appears to be a cause of sport participation. Sport participation appears to be a cause of participation in physical education classes, which in turn appears to be a cause of higher enjoyment. Higher enjoyment appears to be a cause of other motives for participating in physical and leisure activities, as well as a higher score in terms of physical health. The causal diagram provided a graphical representation of the causal relationship between sport participation and better QoL with potential confounders for children and adolescents with heart disease that nearly coincided with the existing literature. Clinical trials should be designed to validate clinical utility of the presented causal diagram.

## 1. Introduction

Since the recommendations for physical activity (PA), recreational sport, and exercise training in pediatric patients with congenital heart disease (ConHD) were published a decade ago (1), a number of studies have assessed the PA level and the reported associations between higher PA levels and better health-related outcomes in this population (2–5). Interestingly, discrepant results exist regarding the differences in PA level between children with ConHD and healthy peers (6–8). The authors of a review on PA modification in youth with ConHD published in 2021 presented that 50% of the analyzed studies showed a lack of significant differences in PA levels between patients and age-matched healthy peers (6). Comparable PA levels of children with ConHD to normal children, however, higher than previously reported by others, were recently observed by authors from Belgium, who speculated that this could be attributed to their policy of motivating and encouraging children to participate in sports (9). Indeed, sport is one of the best investments for promoting PA (10), as it provides medical and psychosocial benefits for children and adolescents (11, 12). Sport participation could be a solution to promote PA in children and youth with ConHD as interventions aimed at improving PA levels in this population did not produce the expected outcomes (13–16). Nevertheless, despite the existence of published evidence- and practice-based guidelines for pediatric cardiac rehabilitation programs (17), as well as recommendations for exercise (18) and examination procedures that aim to ensure safe participation in sports for each individual with ConHD (1, 19–23), and positive consequences of sports participation in this group (24–26), recent studies continue to report instances where patients experience a lack of PA promotion and/or restriction on sport participation by physicians (27–30). In addition, parental overprotection, children's low self-efficacy and fear to exercise, lack of adequate information on PA clearance of the physical education teachers are listed as factors that limit sport participation in youth with ConHD (6, 14, 31).

Reviewing the barriers and facilitators contributing to children sports participation is important (32, 33), so that healthcare providers and parents may be able to enhance and promote sport activities in children with ConHD (26, 29, 34–36). Therefore, we aim to evaluate relationships (particularly in the causal sense) that can be discovered from the questionnaire responses regarding various factors. This can be accomplished using data-driven causal discovery methods, which are intended to search the causal structure [so-called directed causal diagrams, directed acyclic graph (DAG)] from observational data (37, 38). The aims of this pilot study are to discover a causal diagram of sport participation in children and adolescents with heart disease and establish the factors that affect and are affected thereof through the use of questionnaires (treated as observational data). Furthermore, the study aims to qualitatively assess the reliability of the constructed diagram in comparison with existing medical knowledge (addressed in the Discussion).

## 2. Method

### 2.1. Study design and population

The present study is reported in accordance with STROBE guidelines (39). The observational prospective study was conducted from January 2022 to January 2023. The study included a cohort of 121 patients, aged from 8 to 18 years, who voluntarily participated in the study during their routine pediatric follow-up visit. The study eliminated children with (a) impairments that hindered their ability to understand and/or respond to the items in the questionnaires, (b) non-cardiac diseases that prevented them from sport participation, and (c) without confirmed diagnosis. All the interviewed children completed the questionnaires under the supervision of one parent or legal guardian. This study is a part of the research project on assessment of determinants of PA levels in pediatric patients with heart disease.

### 2.2. Questionnaire

The team prepared a questionnaire consisting of closed-ended questions pertaining to demographic data (age, sex, place of residence, number of siblings), clinical cardiac diagnosis (ConHD, cardiac arrhythmia, cardiomyopathy, myocarditis), sport participation status of the patient (actual and before diagnosis with additional information such as type, duration, and frequency of the sport and duration of training session), patient's additional activities with potential influence on participation in sport activities (transport to school, participation in domestic chores, physical education classes, out-of-school additional activities, and spending free time: preferred place and companionship), parental education and sport participation status, and sleep disturbances. Table 1 presents all the data that were collected using the questionnaire.

**Table 1:** Differences between the sport participants and non-sport participants groups.

Variables		Sport participants (*n *= 56, 46%)	Non-sport participants (*n *= 65, 54%)	*p*-value
Age (years)		13 (8–17)	14 (8–17)	0.124
Sex	♀/♂	17 (14%)/39 (32%)	33 (27%)/32 (26%)	0.023
Diagnosis	ConHD	23 (19%)	26 (22%)	0.319
	Cardiac arrhythmia	20 (16%)	20 (16%)	
	Cardiomyopathy	1 (1%)	6 (5%)	
	Myocarditis	12 (10%)	13 (11%)	
Place of residence	City	44 (36%)	43 (36%)	0.130
	Village	12 (10%)	22 (18%)	
Siblings	Yes/No	42 (35%)/14 (11%)	53 (44%)/12 (10%)	0.383
	Number	1 (1–3)	1 (1–6)	0.716
	Younger	24 (20%)	22 (18%)	0.352
	Older	15 (12%)	24 (20%)	
	Younger and older	3 (3%)	7 (6%)	
Parental education	Basic	2 (2%)	4 (4%)	0.347
	Secondary	14 (15%)	20 (22%)	
	Tertiary	28 (31%)	23 (25%)	
Sport participation status before diagnosis	Yes/No	41 (34%)/15 (12%)	22 (18%)/43 (36%)	<0.001
	Strength and power	1 (2%)	1 (2%)	0.775
	Endurance	32 (51%)	18 (29%)	
	Mixed	8 (13%)	3 (5%)	
	Duration (years)	5 (1–10)	5 (1–10)	0.408
	Frequency (days/week)	3 (1–7)	2 (1–7)	0.006
	Duration of training session (min)	60 (30–240)	60 (40–120)	0.816
Transport to school	Active	21 (17%)	23 (19%)	0.107
	Passive/motorized	24 (20%)	37 (31%)	
	Both	11 (9%)	5 (4%)	
Participation in physical education classes	Yes/No	51 (42%)/5 (4%)	30 (25%)/35 (29%)	<0.001
	Frequency (days/week)	3 (1–5)	3 (1–5)	0.822
Participation in out-of-school additional activities	Yes/No	9 (7%)/47 (39%)	10 (8%)/55 (46%)	0.918
	Frequency (days/week)	2 (1–3)	2 (1–6)	0.891
	Duration of one activity (min)	60 (45–240)	60 (20–90)	0.306
Free time – preferred place	Inside	22 (18%)	30 (25%)	0.702
	Outside	31 (26%)	31 (26%)	
	It does not matter	3 (2%)	4 (3%)	
Free time – companionship	With friends	38 (31%)	40 (33%)	0.548
	With family	10 (8%)	17 (14%)	
	Alone	8 (7%)	8 (7%)	
Participation in domestic chores	Never	4 (3%)	7 (6%)	0.286
	<3 days per week	33 (27%)	36 (30%)	
	>3 days per week	13 (11%)	20 (16%)	
	Always	6 (5%)	2 (2%)	
Sleep disturbances	No	38 (31%)	28 (23%)	0.020
	<3 days per week	10 (8%)	17 (14%)	
	>3 days per week	8 (7%)	20 (17%)	
Mother's sport participation status	Yes	26 (21%)	12 (10%)	0.004
	No	17 (14%)	30 (25%)	
	Former athlete	13 (11%)	23 (19%)	
Father's sport participation status	Yes	24 (20%)	16 (13%)	0.043
	No	14 (11%)	29 (24%)	
	Former athlete	18 (15%)	20 (17%)	

### 2.3. Pediatric Quality of Life Inventory 4.0 Core Scales

The Health-related quality of life (HRQoL) was assessed using a Polish version of the Pediatric Quality of Life Inventory 4.0 (PedsQL™) Generic Core Scales questionnaire—a self-report version for children (8–12 years) and adolescents (13–18 years). The questionnaire consists of 23 items in four scales that assess the physical functioning, emotional functioning, social functioning, and school functioning. The items were scored on a 5-point Likert scale from 1 “Never a problem” to 5 “Almost always a problem.” The participants were asked how much of a problem each item has been during the past month. A higher score represented a better HRQoL (40).

### 2.4. Physical Activity and Leisure Motivation Scale—Youth

The Physical Activity and Leisure Motivation Scale—Youth (PALMS-Y) consists of 28 items with seven subscales (mastery, enjoyment, psychological condition, physical condition, appearance, affiliation, and competition/ego) measuring different types of motives for participating in physical and leisure activities. Each subscale consists of four items. The response format for all of the items is a 5-point Likert scale rated from 1 “Strongly disagree” to 5 “Strongly agree,” higher scores reflect the participant's experience of a higher level of that motive for participating in physical and leisure activities (41).

### 2.5. Statistics

The data-driven search of the DAG (causal discovery) was performed using the Greedy Fast Causal Inference (GFCI) technique for continuous variables. The GFCI approach is a combination of and improvement upon two algorithms:

• Fast Causal Inference (FCI) (42), which begins with a complete undirected graph.
• Fast Greedy Equivalence Search (FGES) (43), which starts with an empty graph, and adds iteratively needed edges, and then eliminates unnecessary ones in a graph.

The GFCI method uses the latter one (FGES) to find a “supergraph” (the skeleton of connections), and then the former (FCI) is utilized to prune the “supergraph” to find the final orientations (44).

This method can assess whether the relation is direct and whether there are any latent confounders between the two variables being analyzed.

The whole analysis was performed using Tetrad 6.5.3 software (45). The input data were the table with rows corresponding to the study participants and columns with various variables recorded from the questionnaire (as listed in subsequent lines in Tables 1, 2). All variables were stored as numeric values (so that the technique for continuous variable can be utilized), and the table was prepared in a way that no information regarding the column names and their respective meanings was provided for the analysis, and the order of the data columns and rows did not play any role.

**Table 2:** Differences in PedsQL and PALMS between the sport participants and non-sport participants groups.

Variables	Sport participants (*n* = 56)	Non-sport participants (*n* = 65)	*p*-value
PedsQL			
Total score	78 (35–96)	67 (44–99)	0.002
Physical health	88 (31–100)	72 (31–100)	<0.001
Psychosocial health	78 (37–97)	67 (42–98)	0.029
Emotional functioning	75 (10–100)	63 (10–100)	0.010
Social functioning	95 (30–100)	80 (40–100)	0.092
School functioning	70 (30–100)	63 (20–95)	0.127
PALMS			
Mastery	12.5 (0.0–15.0)	11.0 (3.0–14.0)	<0.001
Physical condition	12.0 (5.0–15.0)	12.0 (3.0–15.0)	0.024
Affiliation	15.0 (3.0–20.0)	15.0 (0.0–20.0)	0.275
Psychological condition	10.0 (4.0–15.0)	9.0 (0.0–15.0)	0.018
Appearance	11.5 (0.0–15.0)	11.0 (0.0–15.0)	0.419
Other's expectations	5.0 (0.0–15.0)	5.0 (3.0–10.0)	0.150
Enjoyment	13.0 (3.0–15.0)	11.0 (0.0–15.0)	<0.001
Competition/ego	8.0 (0.0–15.0)	7.0 (0.0–14.0)	0.001

Then, we used default settings for the GFCI method:

• Cutoff for *p*-value (0.01).
• Penalty discount (2).
• Faithfulness assumed.
• Unlimited length for any discriminating path.
• The complete FCI rule set was not used.
• No bootstraps.
• Ensemble method: highest.

**Figure 1:**
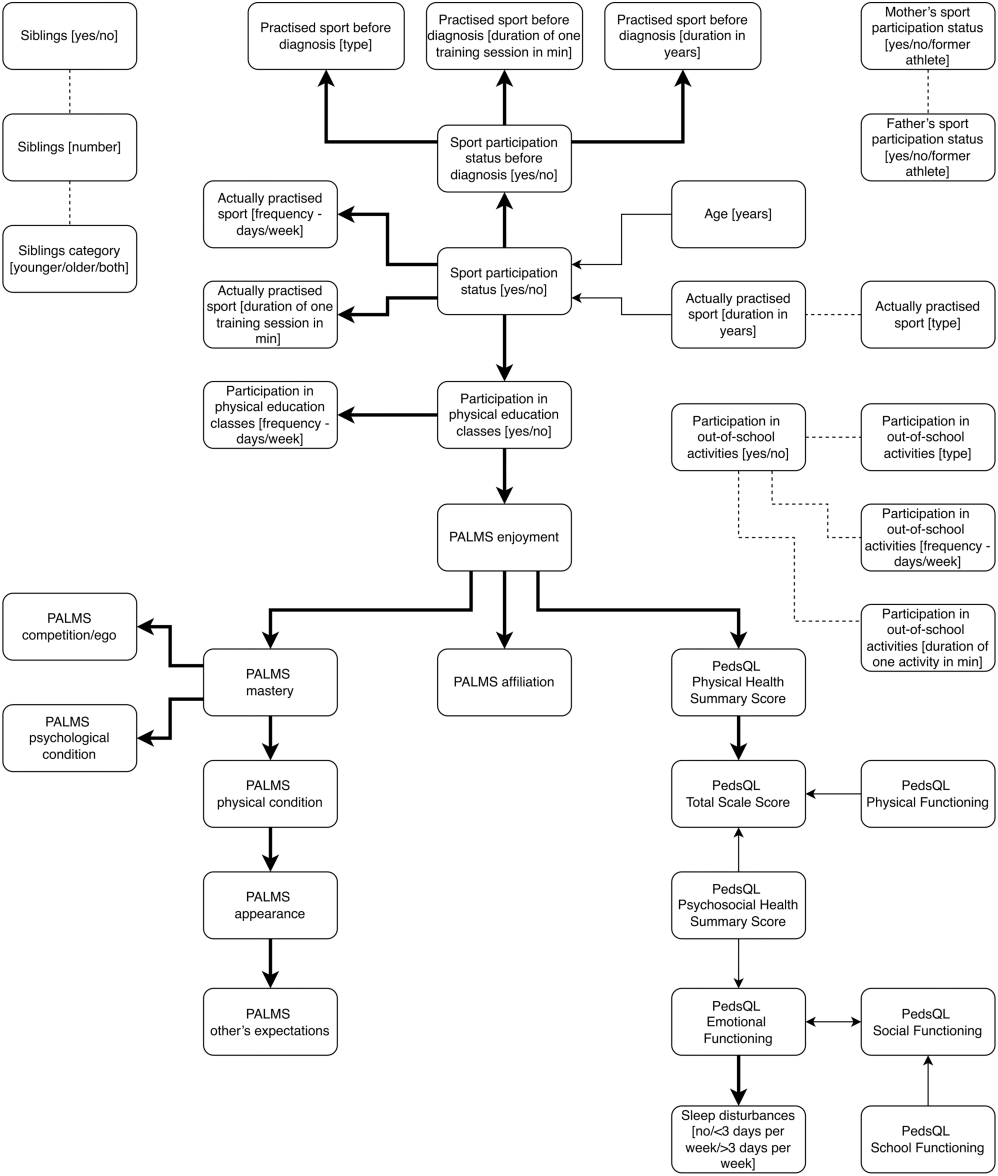
The causal diagram presenting the relationships between the variables collected using the questionnaires. The thickened and solid arrow from one variable to another means the former appears to be the direct cause of the latter, and there is no latent confounder between them. A thin and solid arrow means either the input variable is a cause of the output one or there is an unmeasured confounder. The thin and dashed line represents the case in which one of the following holds: first variable is a cause of another, the opposite relationship, there is an unmeasured confounder, both the first relation with the unmeasured confounder, or both the second relation with the unmeasured confounder.

As a result, the output graph was created, and hidden column names were back uncovered in order to produce Figure 1.

The Shapiro–Wilk test was used to test the data distribution for normality. The *U*-Mann–Whitney non-parametric test was utilized to compare the medians of the two groups (unpaired case). The *χ*^2^ test was used to compare the frequencies between groups. The level of statistical significance was set at *p* < 0.05 (STATISTICA 13, StatSoft Inc., Tulsa, OK, USA).

## 3. Results

There were 121 completed questionnaires received from Caucasian patients (*n *= 50 females) diagnosed with heart disease with a median age of 13 years (range: 8–17). The descriptive statistics of all data that were collected from the questionnaires are presented in Table 1. Table 2 presents the PedsQL and PALMS scores. There were 56 (46%) sport participants and 65 (54%) non-sport participants. A total of 22 (18%) patients had stopped their participation in sport due to their diagnosis. Among the groups of sport participants and non-sport participants, there were five and 25 patients, respectively, who had medical exemptions from participating in physical education classes.

The study found that sport participants had a higher proportion of boys (*p* < 0.05) and children who engaged in sports prior to diagnosis (*p* < 0.001), who participated in physical education classes (*p* < 0.001), with a lower number of nights with sleep disturbances (*p* < 0.05), and parents that participated in sport activities (*p* < 0.05) than the non-sport participants. Sport participants practiced endurance sports (*n *= 45), strength and power sports (*n *=* *4), and mixed sports (*n *=* *7). They practiced a sport with a median of three times per week (range: 1–7). In addition, the median duration of one training session was 60 min (range: 30–240). The reasons for participation in sport were as follows: because I like it/it is my passion (*n *=* *41), to take care of the health and body (*n *=* *29), to spend time with friends (*n *=* *26), to relieve emotions or stress (*n *=* *16), to spend time with parents (*n *=* *12), and because my parents encourage me (*n *=* *2).

Sport participants presented significantly higher values of HRQoL in total score, physical health, psychosocial health, and emotional functioning. In addition, they exhibited significantly higher values in PALMS-Y subscales: mastery, physical condition, psychological condition, enjoyment, and competition/ego (see Table 2).

Figure 1 presents the causal diagram as a result from the GFCI method, estimated in a data-driven scenario. From the perspective of the study subject, some relationships should be particularly indicated:

(i) Age appears to be a cause of sport participation in pediatric patients with heart disease. Sport participants were younger.
(ii) Sport participation appears to be a cause of participation in sport activities prior to diagnosis and participation in physical education classes—this appears to be a cause of higher intrinsic motive—enjoyment.
(iii) Higher patients’ enjoyment appears to be a cause of higher affiliation and mastery motives but also higher physical health score.
(iv) Both higher physical and psychosocial health scores appear to be causes of better HRQoL (total PedsQL score).
(v) Higher psychosocial health score appears to be a cause of higher emotional functioning.
(vi) Lower emotional functioning appears to be a cause of sleep disturbances.

The variables included in the questionnaire that were found to be causally unrelated with other variables were sex, diagnosis, place of residence, parental education, reasons for participation in sport, place and/or companionship for spending free time, transport to school, and participation in domestic chores.

## 4. Discussion

In this pilot study, the idea was to discover the causal diagram of factors that may affect and be affected by sport participation in children and adolescents with heart disease and to assess the reliability of the diagram by comparing it with the existing literature. Based on the diagram, sport participation appears to be a cause of participation in physical education classes, and this practice appears to be a cause of higher enjoyment. Higher patients’ enjoyment appears to be a cause of other motives for participating in physical and leisure activities, as well as a higher physical health score. Importantly, emotional functioning appears to be a cause of sleep disturbances. Among the analyzed variables, only age appears to be a cause of sport participation, however, with unmeasured confounder(s). It is worth mentioning that other factors that may influence on the likelihood of sport participation in pediatric patients with heart disease such as sex, diagnosis, or parents’ sport participation status were found to be causally unrelated with the sport participation status of the patients. The heterogeneity of the group may have impacted these results.

The GFCI method employed in our study successfully identified a graph of sport participation in pediatric patients with heart disease that nearly coincided with the existing literature. There are several original studies on sport participation in pediatric patients with ConHD, mostly assessing its association with QoL. The correlation between beliefs in self-efficacy and involvement in sport and PA was shown to be stronger in adolescents than the severity of the disease (46). Higher self-efficacy, male gender, and participation in physical education classes were associated with a higher likelihood of engagement in PA (34). Patients who exercised more often (school classes or fitness centers) were more likely to participate in leisure or competitive sports (25). Participation in sports was associated with improved exercise capacity, emotional, psychological, and social wellbeing, and generally with a higher QoL (47, 48). An improvement in health status has been observed after a 3-day multi-sports camp (49). Participating in recreational sport encouraged patients to be more intrinsically motivated to engage in PA—patients emphasized their enjoyment of PA as a primary source of motivation (26). The findings from existing literature suggest the occurrence of closed-loop interaction between higher self-efficacy and/or higher intrinsic motivation and higher involvement in sport and PA in pediatric patients with ConHD. Understanding the patient's motivation for PA or sport can be helpful to a clinician or a physical therapist in order to stimulate participation in PA and/or identify suitable forms of activity. To assess an individual's goal-oriented motivation, the Physical Activity and Leisure Motivation Scale was developed (41). The scale measures eight types of motivation—enjoyment and mastery factors, significantly higher in group of sport participants in our study, can be considered as intrinsic motivation, while the other six factors describe extrinsic motivation based on the self-determination theory (50).

There is an urgent need for global action aimed at decreasing levels of insufficient activity in both healthy children and pediatric patients with chronic conditions and disability (51). Proposed interventions aimed at improving PA levels in children and youth with ConHD did not produce the expected outcomes (13–16). Learning causal diagram discovered *a priori* with longitudinal data providing extensive prior knowledge may be a helpful technique in establishing the kind and dose of sport activities as a potential solution for promoting PA in this population.

Causal diagrams based on observational data have been utilized in Alzheimer's pathophysiology (52). The authors constructed a “gold-standard” causal structure graph based on the existing literature. Then, they applied three algorithms to discover this causal structure from observational data. FGES, the method used in the presented study, managed to (almost perfectly) recover the “gold-standard” graph. In addition, the authors provided a detailed guideline on how causal discovery algorithm can be applied to discover high-quality causal relationships (52). Recently (2023), Huie et al. (53) discussed chronic low back pain as a “Big Data” problem and identified how data-driven method that uses artificial intelligence and high dimensional datasets (constraint-based: FCI and score-based: FGES algorithms) to identify causal structures may help better understand causal factors for low back pain, guide clinical practice, and improve outcomes.

The causal diagram was presented as solely the data-driven output of the GFCI method, without supplementing it with any specific medical knowledge or experience-based intuition. It seemed that some relations had not been “detected” by a method (we were aware of such a possibility prospectively), even if simple comparisons (presented in Table 1) are found as significant. Perhaps, these relationships may be further emphasized with a similar analysis conducted on a larger population. As with all studies, there are a number of limitations to consider. These include the limited dataset used for identifying DAG (no sensitivity analysis could be conducted), heterogeneity of a small study group, and the failure to differentiate between different types of sports, i.e., competitive or recreational/leisure. The inclusion of participants across a wide age range in the study group should be also considered as a study limitation—drop out from sports is increasing with increasing age during childhood and adolescence (54). The directed edges in the discovered DAG are being interpreted causally. Accordingly, the edge Age → Sport participation status in the graph can be interpreted as age causally influences the sport participation status in pediatric patients with heart disease. The results of the direct comparison showed that sport participants were younger; however, as this relation is not separated in the DAG, it does not inform regarding the extent to which a change in age would causally affect sport participation status.

One advantage of conducting this pilot study is that it will pave the way for future research involving a larger population of pediatric patients with heart disease and will include more factors that have the potential to affect and/or might be a consequence of sport participation, such as PA level, obesity status, cardio-respiratory function, health-related physical fitness, cardiac module of PedsQL, and parental support. Then the objective would be to: (i) identify the appropriate set of variables to minimize bias due to potential confounding associations and (ii) to generate hypotheses to be tested (as interventions in the causal sense) in future trials.

## 5. Conclusion

In conclusion, causal diagram provided a graphical representation of the causal relationship between sport participation and better QoL with potential confounders for children and adolescents with heart disease that nearly coincided with the existing knowledge. However, it is important to exercise caution in interpreting these findings, as there are certain limitations that have been mentioned. Clinical trials should be designed to validate the clinical utility of the presented causal diagram. The optimal dose of PA (55) and promotion strategies still remain a challenge. Pediatric cardiologists should propose appropriate sport activities for children and adolescents with heart disease as a strategy for promoting active lifestyles to improve QoL.

## Data Availability

The raw data supporting the conclusions of this article will be made available by the authors, without undue reservation.
